# 

**Published:** 1899-06

**Authors:** 


### CONTENTS.

ORIGINAL COMMUNICATIONS—

The Treatment, Medical Complications and Sequelæ of Typhoid Fever. By H. A. Hare, M.D., Philadelphia, Pa. .... 217

The Endoscopic Treatment of Chronic Urethritis. By W. L. Champion, M.D., Atlanta, Ga. .... 236

Seven Cases of Diphtheritic Croup—Two Aborted by Antitoxin and Five Cured by Antitoxin and Intubation. By R. M. Harbin, M.D., Rome, Ga. .... 241

The Treatment of Some Cases of Cancer of the Skin. By M. B. Hutchins, M.D., Atlanta, Ga. .... 248

SOCIETY REPORTS—

The Clinical Society of Maryland .... 254

EDITORIAL NOTES AND COMMENTS—

"As Ye Sow that Shall Ye Also Reap" .... 265
"Husa" Sucker .... 267
Mummified Meat .... 268

CONTENTS CONTINUED .... vi

